# RESIDENTIAL PREFERENCES FOR CURRENT AND FUTURE RESIDENTIAL ENVIRONMENTS AMONG KOREAN MIDDLE-AGED ADULTS

**DOI:** 10.1093/geroni/igae098.1669

**Published:** 2024-12-31

**Authors:** Eunju Lee, Kyungmin Kim

**Affiliations:** Seoul National University, Seoul, Republic of Korea; Seoul National University, Seoul, Republic of Korea

## Abstract

Midlife adults often plan for life after retirement, forming their own preferences for where to live after retirement. Their residential preferences for life after retirement may be different from the past as they consider their well-being in later life. This study compared middle-aged adults’ preferences for residential features between current and future residential environments (i.e., past and current residential preferences) and examined how differences in preferences for current and future residential features were associated with health and social characteristics. A sample of 1,651 Korean middle-aged adults (aged 49–64) rated their preferences for each of 12 residential features (e.g., proximity to family/relatives, low living expenses, accessibility to health services) regarding their current and future residential environments, separately. Respondents considered “house price” and “safe and clean environments” most important for both current and future residential environments, while “accessibility to health services” and “opportunity for leisure activities” were more important for future residential environments. Regression results showed that respondents with poorer health considered features that help them compensate for health decline more important, while those who contacted friends/neighbors more frequently and participated in more cultural activities considered features that help them maintain their social interactions more important for future residential environments than the past. Across residential features, respondents with poorer health showed lower similarity in overall preferences between current and future residential environments. Our findings suggest the importance of identifying various demands for residential features after retirement to support successful adaptation in later years.

